# Severe intraventricular hemorrhage causes long-lasting structural damage in a preterm rabbit pup model

**DOI:** 10.1038/s41390-022-02075-y

**Published:** 2022-05-03

**Authors:** Olga Romantsik, Emily Ross-Munro, Susanne Grönlund, Bo Holmqvist, Anders Brinte, Erik Gerdtsson, Suvi Vallius, Matteo Bruschettini, Xiaoyang Wang, Bobbi Fleiss, David Ley

**Affiliations:** 1 Department of Clinical Sciences Lund, Division of Pediatrics, Lund University, Skåne University Hospital, 21185 Lund, Sweden; 2 School of Health and Biomedical Sciences, STEM College, RMIT University, Bundoora, 3083 VIC Australia; 3 ImaGene-iT AB, 22363 Lund, Sweden; 4 Centre of Perinatal Medicine & Health, Institute of Clinical Sciences, Department of Obstetrics and Gynecology, Sahlgrenska Academy, Gothenburg University, 40530 Gothenburg, Sweden; 5 Henan Key Laboratory of Child Brain Injury and Pediatric Clinical Research Center, Institute of Neuroscience and Third Affiliated Hospital of Zhengzhou University, Zhengzhou, China; 6 Université de Paris, NeuroDiderot, Inserm, 75019 Paris, France

## Abstract

**Background:**

Intraventricular hemorrhage causes significant lifelong mortality and morbidity, especially in preterm born infants. Progress in finding an effective therapy is stymied by a lack of preterm animal models with long-term follow-up. This study addresses this unmet need, using an established model of preterm rabbit IVH and analyzing outcomes out to 1 month of age.

**Methods:**

Rabbit pups were delivered preterm and administered intraperitoneal injection of glycerol at 3 h of life and approximately 58% developed IVH. Neurobehavioral assessment was performed at 1 month of age followed by immunohistochemical labeling of epitopes for neurons, synapses, myelination, and interneurons, analyzed by means of digital quantitation and assessed via two-way ANOVA or Student’s *t* test.

**Results:**

IVH pups had globally reduced myelin content, an aberrant cortical myelination microstructure, and thinner upper cortical layers (I–III). We also observed a lower number of parvalbumin (PV)-positive interneurons in deeper cortical layers (IV–VI) in IVH animals and reduced numbers of neurons, synapses, and microglia. However, there were no discernable changes in behaviors.

**Conclusions:**

We have established in this preterm pup model that long-term changes after IVH include significant wide-ranging alterations to cortical organization and microstructure. Further work to improve the sensitivity of neurocognitive testing in this species at this age may be required.

**Impact:**

- This study uses an established animal model of preterm birth, in which the rabbit pups are truly born preterm, with reduced organ maturation and deprivation of maternally supplied trophic factors.
- This is the first study in preterm rabbits that explores the impacts of severe intraventricular hemorrhage beyond 14 days, out to 1 month of age.
- Our finding of persisting but subtle global changes including brain white and gray matter will have impact on our understanding of the best path for therapy design and interventions.

## Introduction

Intraventricular hemorrhage (IVH) occurs in up to 45% of preterm infants born below gestational age 26 weeks, with severe IVH (≥3 grade) occurring in nearly 30% of those very fragile infants.^1–3^ These numbers have increased as survival of the smallest infants, 22–23 weeks, improves.^4,5^ Of these infants with severe IVH (≥3 grade), up to 60% will go on to develop post-hemorrhagic ventricular dilation (PHVD).^1,6^ We have no treatments for IVH, and infants born preterm and affected by IVH have a higher risk for impaired neurodevelopment^4,7,8^ compared to their age-matched non-IVH affected peers and this is especially true for infants who go on to develop PHVD.^9–14^

IVH arises in the germinal matrix, a layer of immature neuronal and glial precursors adjacent to the ependymal lining of the ventricles interwoven with a dense network of delicate blood vessels. These vessels have no structural support, and lack autoregulation, which makes them susceptible to fluctuations of vascular flow. The risk factors for IVH include immaturity (gestational age, birth weight), vascular fluctuations (hypotension, need for resuscitation), and inflammation (interleukin 1B polymorphism,^15^ and chorioamnionitis.^16^) The development of therapies for IVH-related brain injury is stymied by a lack of well-characterized representative animal models.

Models of IVH across species have revealed mechanisms potentially involved in its pathophysiology, reviewed in ref. ^17^ including the distribution of extracellular hemoglobin within white matter (WM), toxicity of blood product degradation, onset of neuroinflammation characterized by microglia and astrocyte activation and infiltration by systemic immune cells, the death of neuronal and glial cells, and arrest of pre-oligodendrocyte maturation. However, most IVH models utilize pups delivered at term with correspondingly mature physiology but with varying degrees of brain maturity when compared to the human infant, ranging from preterm (rodents) to term (piglets, dogs).^17,18^ The lack of preterm delivery in those models omits several important aspects of preterm birth, such as respiratory instability, immaturity of the coagulation system, a deficit of trophic factors, and the impact of hemorrhage into the immature brain parenchyma.^17^

One of the few small animal models of IVH that incorporates preterm birth is the preterm rabbit pup model,^19–21^ which is used in the current study. Preterm rabbit pups exhibit many aspects of prematurity relevant for human preterm infants, including smaller lungs with a reduced alveolar surface and lower expression of surfactant proteins,^22,23^ the propensity to development of necrotizing enterocolitis-like disease,^24^ renal immaturity,^25^ and reduced levels of trophic growth factors such as insulin-like growth factor-1.^26^ The preterm rabbit brain development at post-conceptional day 29 arguably corresponds to brain development in humans at gestation week 24–25,^20,27,28^ the period of peak vulnerability to IVH.

Across all models of IVH, most studies report on short-term outcomes (<14 days), and there is a lack of neurobehavioral testing in preterm models. Thus, to fill this gap in our knowledge, we aimed to use our established model of IVH in preterm rabbit pups and characterize out to 1 month of age (equivalent to a 1-year-old human) survival, neurobehaviors, neuropathology, and cortical development.

## Materials and methods

### Animals

The study was approved by the Swedish Animal Ethics Committee in Lund (dnr. M 2-16). We used the preterm rabbit pup model of glycerol-induced IVH as previously described.^20,21^ Seventy-seven preterm rabbit pups of both sexes from 12 different litters were included in the study. Detailed methods can be found in the Supplementary Methods. A half-breed between the New Zealand White and Lop was used (Christer Månsson, Löberöd, Sweden). Pups were delivered via cesarean section at post-conceptional day 29 (term = 32 days). At 3 h of age, all pups received intraperitoneal (i.p.) injection of 50% (*v*/*v*) sterile glycerol (6.5 g/kg; Teknova, Hollister, CA) to induce IVH. Thereafter, pups were randomly allocated (https://www.random.org/) to a wet-nurse doe for the remainder of the experiment. The study endpoint was PND33, which corresponds roughly to brain development of a year-old toddler.^29^

### IVH and PHVD detection

The presence and severity of IVH were evaluated by high-frequency ultrasound (HFU) (VisualSonics Vevo 2100, VisualSonics Inc., ON, Canada) using an MS-550D 40 MHz transducer at PND1 and PND2. Pups with IVH (any grade) as determined by HFU (HFU was done by O.R.) were assigned to the IVH group and those without detectable IVH were used as controls. To confirm the presence of PHVD, an ex vivo brain HFU was also performed directly after termination via an artificial ultrasound window created by shaving the skull and making a short skin mid-sagittal incision and making a careful osteotomy (approximately 3 mm), taking care to prevent damage to the underlying dura. The obtained calvarial opening was irrigated with sterile saline to wash out tissue microparticles.

### Sex determination

To detect the male sex, polymerase chain reaction for the detection of SRY (specific region of the Y chromosome) sequences was used; details of the PCR conditions and the primer sequences are found in the Supplementary Methods.

### Neurobehavioral examination

Neurobehavioral testing was performed between PND29 and 32 as previously described^20,30,31^ by an investigator blinded to the group of the pup. There was no visible differences in general animal performance that allowed us to discriminate between the groups. The motor examination included muscle tone and strength, gait, and righting reflex. The neurocognitive testing included open field (OF) test and the object recognition test (ORT). The ORT included a familiarization phase and a trial phase, which was spaced by an interest interval of 5-, 30-, and 240-min on testing days 2, 3, and 4, respectively. The specific details of the tests, arena, and the paradigms in full are found in the Supplementary Methods section. The room temperature was set at 21 °C and works undertaken as previously in rabbit pups at earlier time points.^31,32^ Data were analyzed with the Video Tracking Software (SMART, Panlab SL, Barcelona, Spain). The authors were blinded to animal group assignment throughout testing, data gathering, and analysis.

### Tissue collection

Tissues were collected and processed as previously^31^ and as described in detail in the Supplementary Methods. In brief, brains were transcardially perfused and then post-fixed for 24 h in 4% paraformaldehyde before being processed to paraffin and cut at 5 µm.

### Immunohistochemistry (IHC)

Sections were prepared and stained using well-established protocols as previously^31^ and described in detail in the Supplementary Methods. In brief, sections were rehydrated and antigen retrieval was performed with citrate buffer, pH 6 + 0.04% Tween 20, at 95 °C for 20 min followed by blocking with 0.1 M phosphate-buffered saline (PBS) containing 0.05% Triton X-100 (TX) and 1% bovine serum albumin (PBS-BSA-TX) for 30 min and then the application of primary antibodies listed in Table 1 diluted in PBS-BSA-TX and applied for 16 h, at 4 °C in a humidified chamber: As antibody specificity controls, in adjacent sections the primary antibody incubation was excluded. Sections were then incubated with horseradish peroxidase (HRP)-conjugated secondary antibodies, see Table 1, for 30 min at room temperature and diaminobenzidine (DAB, 0.5 mg/ml) plus hydrogen peroxidase (0.1%) for 10 min at room temperature to reveal the antigen–antibody binding. Sections were dehydrated and coverslipped in Pertex (Histolab, Gothenburg, Sweden).

**Table 1:** Antibodies used in the study.

Antigen	Species and antibody type	Product identifier
Immunohistochemistry		
Neuronal nuclear antigen (NeuN)	Mouse monoclonal (1:100)	MAB377, Millipore, Ternecula, CA, USA
Synaptophysin	Mouse monoclonal (1:40)	Clone Sy38, ab8049, Abcam, Cambridge, UK
Myelin basic protein (MBP)	Mouse monoclonal (1:50)	Clone SMI94, 836504, BioLegend, San Diego, CA, USA
Ionized calcium-binding adapter molecule 1 (IBA 1)	Rabbit polyclonal (1:1000)	019-19741, FUJIFILM Wako, Japan
Glial fibrillary acid protein (GFAP)	Chicken polyclonal (1:750)	ab4674, Abcam, Cambridge, UK
Mouse IgG	Goat polyclonal (1:1)	MP-7452, Vector Oxfordshire, UK
Chicken IgY	Donkey polyclonal (1:500)	703-585-155 Jackson IR, West Grove PA, USA
Immunofluorescence		
Chicken ovalbumin upstream promoter transcription factor-interacting protein 2 (CTIP2)	Rat monoclonal (1:500)	ab18465, Abcam, VIC, Australia
N-terminal EF-hand calcium binding protein 1 (NECAB1	Mouse polyclonal (1:500)	PA5-54849, Thermo Fisher, VIC, Australia
Parvalbumin (PV)	Mouse monoclonal (1:250)	PV235, Swant, Burgdorf, Switzerland
Perineuronal net acetylgalactosamines	Wisteria Floribunda lectin (WFL, non-antibody based) (1:500)	B-1355, Vector laboratories via Abacus, QLD, Australia
Anti-mouse IgG	594 goat polyclonal (1:200)	A11034, Thermo Fisher, VIC, Australia
Biotin	Streptavidin 488 conjugate (1:1000)	S11223, Thermo Fisher, VIC, Australia
Anti-rat IgG	594 goat polyclonal (1:500)	A11007, Thermo Fisher, VIC, Australia
Anti-mouse IgG	594 goat polyclonal (1:500)	A11011, Thermo Fisher, VIC, Australia

### Immunofluorescence (IF)

A standardized IF protocol was conducted as previously described^33^ and described in detail in the Supplementary Methods, including no primary antibody controls. In brief, slides were rehydrated and antigens were retrieved with citrate buffer (10 mM, pH 6.0) at 95 °C for 20 min. Sections were then exposed for 20 min to a 0.1% Sudan Black block diluted in 70% EtOH, as previously,^34^ and then blocked further in 5% normal goat serum 0.2% Triton X-100 for 1 h at room temperature. Slides were incubated in humidified chambers overnight at 4 °C with the primary antibodies and then the secondary antibodies listed in Table 1. Sections were then stained with 4,6-diamidino-2-phenylindole and coverslipped.

### Image acquisition and quantification

We initially verified that there was no labeling in the antibody-omitted specificity control sections. Analyses were conducted by investigators blinded to the treatment groups. Full details of the methods can be found in the Supplementary Methods section. Sections were scanned to obtain digital images and the region of interest (ROI) extracted with resolution of scale fixed across sections, regions, and analysis.

NeuN-positive cells were calculated with Fiji by the default auto-threshold segmentation method and watershed separation following primary smoothing or were manually counted if the separation of cells was not achieved. The number of identified cells was divided by the whole area for neuron density assessment.

The area coverage for glial fibrillary acid protein (GFAP), synaptophysin, and myelin basic protein (MBP) labeling was calculated using a fixed threshold (relative to background) and the relative area (positive area/overall area) was calculated.

Directionality and organization of the MBP staining was analyzed using Fiji (10.1088/1758-5090/aa6204). MBP was assessed in the middle third of a fixed-width image of the cortical plate to focus on the region with the greatest number of ascending fibers.

For interneuron analysis, upper and lower cortical regions were defined as layers I–III and IV–VI, respectively, as described previously.^33^ Immunoreactive cells were counted manually by a blinded observer using the CellSens Dimension counter tool. PV+ cells were identified as having a defined cell body within the section, and WFL+PNN+ were defined as an entire halo encompassing a PV+ interneuron.

The mean of the left and right regions for each analysis and ROI was used for statistical analysis.

### Statistical analysis

Kaplan–Meier curve with a Gehan–Breslow–Wilcoxon test was used for survival analysis. For neurobehavioral testing, a one-tailed *T* test was employed based on the expected direction of change from previous studies,^1,2^ and analyses were performed using SPSS (PASW Statistics 18, IBM, Deutschland GmbH).

For cortical layering analysis, an unpaired Student’s *t* test assessed treatment (presence of IVH) vs control effect on total cortical depth (Layers I–VI). Grouped data of layer depth (I–III, VI, V–VI; IVH vs control) and comparisons across regions for staining of cell types with IHC were analyzed using two-way analysis of variance (ANOVA), and when statistical significance was attained for treatment (presence of IVH) vs control (no IVH), post hoc analysis was performed with Sidak’s multiple comparison using alpha = 0.05. For IHC analyses, including cortical layers, perineuronal net (PNN), interneuron, and myelin, organization statistics were undertaken with GraphPad Prism (9.0, San Diego, CA) and post hoc analysis was also performed with Sidak’s multiple comparison using alpha = 0.05. Results are shown as mean (standard deviation) or median (interquartile range or confidence interval 95%). For all analyses, *p* < 0.05 was indicative of statistical significance.

## Results

**Fig. 1:**
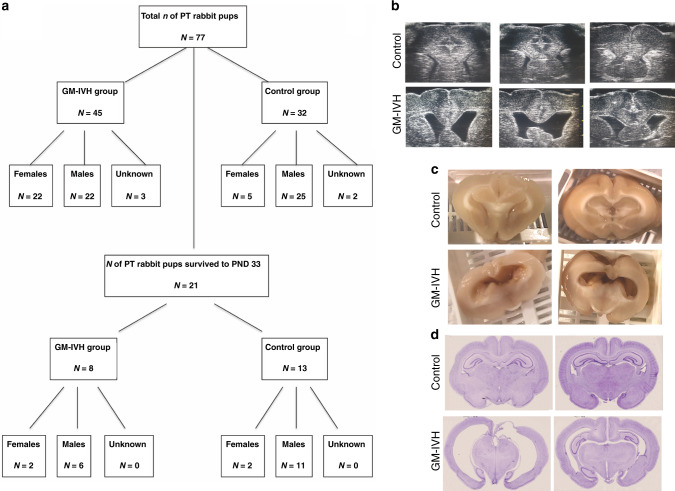
Flow chart of the study and overall disturbance to brain structure. Number of rabbit pups in IVH with PHVD and control groups according to sex (**a**). The impact of IVH was visible via (**b**) high-frequency ultrasound ex vivo at PND33 in which coronal sections were obtained in a rostral–caudal direction; (**c**) at the macroscopic examination; and (**d**) with hematoxylin–eosin stains of coronal sections (scale bar = 25 mm).

As summarized in Fig. 1a, 77 preterm rabbit pups (female = 27, male = 45, unknown = 5) from 12 litters were included in this study who were cross-fostered to a total of ten wet nurses. In total, 88 pups were delivered, but 4 were born dead and 7 died between birth and before 3 h of age when glycerol was administered. All pups received glycerol and 58% developed IVH as assessed with ultrasound. Twenty-one preterm pups (=27%) survived to the endpoint of the study (PND33). Of those 21 preterm pups, 8 (=38%) (females = 2) had IVH and developed PHVD and 13 (=62%) (females = 2) were used as controls (Fig. 1a). Following termination of the pups at PND33, we confirmed the development of various degrees of PHVD in all 8 animals in the IVH group (examples in Fig. 1b–d). One pup in the IVH group had a considerably milder PHVD and was therefore excluded from all subsequent analyses; this pup was male and had otherwise regular post-natal growth. All 13 animals in the control group had a normal brain ultrasound finding (representative images in Fig. 1b).

### Pups with IVH had reduced survival rates

**Fig. 2:**
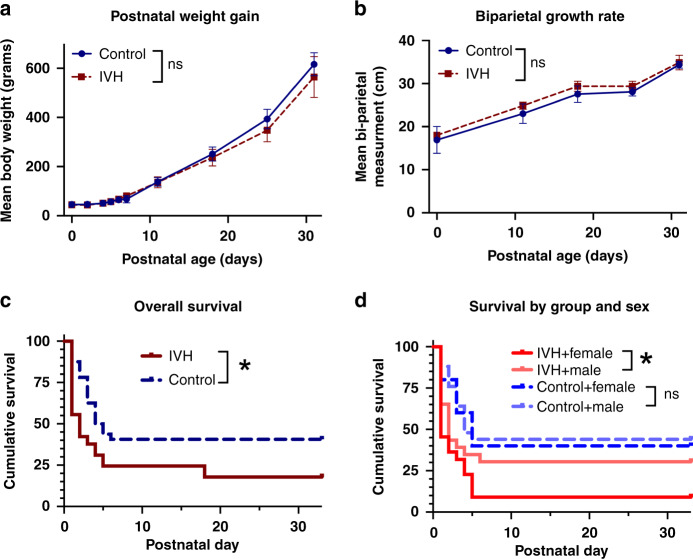
Postnatal growth and survival of rabbit pups. There were no differences in somatic growth indices (**a**, **b**) between all pups with IVH and controls or when analyzed by sex (data not shown). IVH was associated with a significant decrease in pup survival (**c**). Survival in female preterm pups was close to a significantly lower level compared to male preterm pups (*p* = 0.05; **d**). Fewer female preterm pups with IVH survived until the study endpoint compared to the preterm male pups (*p* = 0.015; **d**). Data are shown as median +/− 95% CI, group differences evaluated with two-way ANOVA (**a**, **b**), and survival illustrated with Kaplan–Meier curves (**c**, **d**). Control, *n* = 13 (female = 2); IVH, *n* = 8 (female = 2); *p < 0.05.

There were no differences in birth weight, bi-parietal measurement at birth, and in postnatal growth between the groups (Fig. 2a, b) or by sex for each treatment (data not shown due to small *n*). Survival was drastically diminished in pups with IVH compared to the controls (17.8 vs 40.6 %, *p* = 0.004, Gehan–Breslow–Wilcoxon test; Fig. 2c). Survival in female pups (control and IVH) was significantly lower compared to male pups (control and IVH) (*p* = 0.044, Gehan–Breslow–Wilcoxon test; Fig. 2d). Fewer female preterm pups with IVH survived until the study endpoint compared to the preterm male pups (*p* = 0.013; Fig. 2d).

### IVH had no impact on the assessed neurodevelopmental tests

At PND 29–32, all surviving preterm pups in both IVH and control groups were exposed to neurobehavioral assessment. It was impossible to distinguish phenotypically the preterm pups with IVH/PHVD from the controls: all the surviving pups managed to feed independently, moved freely within the cages, and exhibited no differences when assessed for muscle tone and righting reflex (data not shown). The gait and coordination examination on a 60° inclined slope revealed no differences between the IVH and the control groups (mean (SD): 20.2 ± 11.2 s vs 21.5 ± 15.4 s, *p* = 0.8).

During the OF test with a duration of 5 min, both groups of preterm rabbits spent nearly half of the testing time in the peripheral zone with their body touching the wall (mean (SD) time was 141.5 ± 92 s vs 124.3 ± 93 s, for the IVH and control groups, respectively, *p* = 0.7). Entries into the central zone did not differ by groups as 4 IVH (53%) and 7 controls (53%) entered the central zone of the arena and there were no differences in the exploration time in the central zone between the groups (mean (SD) time was 10.4 ± 11 s vs 8 ± 16 s, for the IVH/PHVD and control groups, respectively, *p* = 0.7). There were no differences in global activity and in total covered distance between the IVH/PHVD group and control groups (mean (SD) distance was 332.16 ± 332.2 cm vs 219.1 ± 286.2 cm, respectively; *p* = 0.5). There was no difference in median latency time to escape from peripheral to the central zone between the groups, being a mean (SD) 64.3 ± 64.3 s vs 86.2 ± 96.0 s for the IVH and control groups, respectively; *p* = 0.6).

There were also no differences in the ORT test: both groups of animals exhibited comparable exploratory behavior with similar exploration times on 3 consecutive examination days. Both groups recognized the old and the new object in the 5-, 30- and 240-min interval without differences between the two groups (Table 2). Two animals (one for each group) were excluded from the ORT analysis: both animals froze at the starting point for the whole 5-min testing period.

**Table 2:** Exploration times and novel object recognition ratios in the ORT.

Exploration time (min)	GM-IVH (*N* = 7)								Control (*N* = 12)							
	Mean	SD	Min	Max	Median	25th	75th	*p* value	Mean	SD	Min	Max	Median	25th	75th	*p* value
Exploration time (min)																
5 min (s)	19.1	14.3	6.5	47.4	11.1	9.6	24.1	0.021	13.1	8.9	0	34.2	11.6	8.0	17.3	0.028
240 min (s)	15.1	9.9	1.95	32.2	12.1	9.1	21.9	0.028	17.8	5.7	7.7	27.6	17.4	14.3	22.9	0.002
Novel object recognition ratios																
5 min	0.47 (−0.07; 0.91) 0.35 (−0,27; 0.84)								0.56 (−1; 1) 0.52 (0.16; 0.87)							
240 min																

Data for novel object recognition ratios are displayed as median with interquartile ranges.

### IVH caused differences in myelin organization

**Fig. 3:**
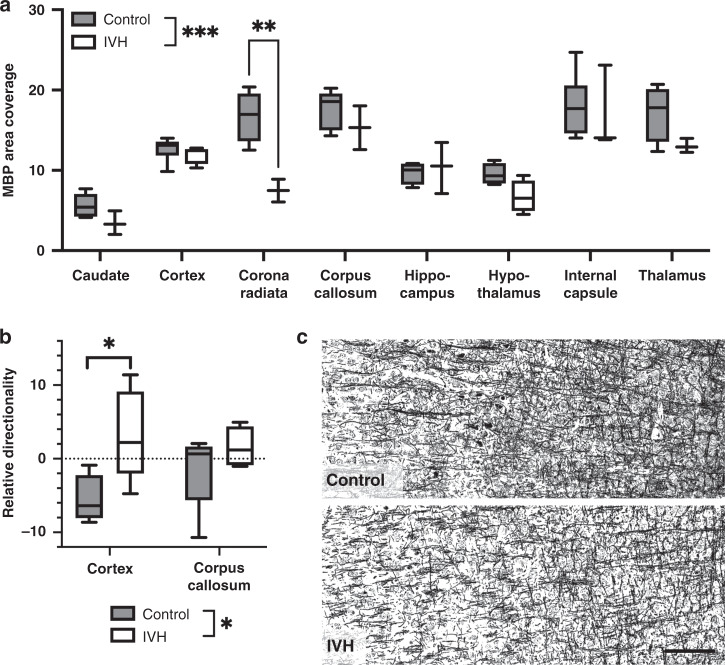
Myelin area coverage and organization were significantly altered in pups with IVH and PHVD. Pups with IVH and PHVD had a reduction of MBP area as compared to controls (**a**) and an altered organization of MBP stained fibers (**b**) as shown in the representative photomicrographs of the cortical MBP staining in (**c**) (scale bar = 20 μM). Data shown are median ± 95% CI. Control, *n* = 6, IVH, *n* = 6. Evaluated with two-way ANOVA, *p < 0.05; **p < 0.01; ***p < 0.001.

The IVH group animals had a reduced density of myelin immunoreactivity (MBP staining) across the eight examined regions compared to the controls (*p* = 0.002, two-way ANOVA). This was revealed to be specifically apparent in the corona radiata compared to the controls in the post-test (*p* = 0.011; Fig. 3a). Furthermore, we analyzed the organization (directionality) of the MBP staining in the cortex and corpus callosum and observed significant effects of IVH (*p* = 0.0146, two-way ANOVA) that in the post-test were specifically apparent in the cortex (*p* = 0.026, Fig. 3b, c).

### IVH reduces the numbers of mature neurons and synaptophysin area coverage and alters the development of the cortical layers

**Fig. 4:**
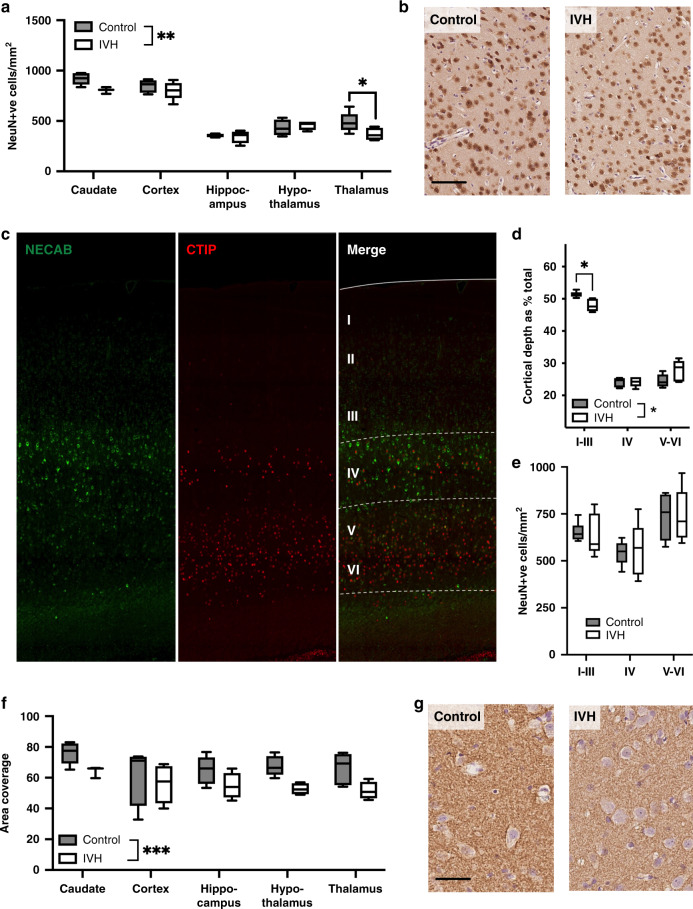
NeuN-positive cell number and synaptophysin coverage is reduced and cortical organization altered in pups with IVH. Pups with IVH had a reduced number of NeuN-positive cells as compared to controls (**a**) shown in a representative micrograph of the thalamus (**b**) (scale bar = 100 μM). Cortical layers were demarcated using staining for CTIP2 and NECAP and compared between pups with IVH and controls (**c**). There was a reduction in the depth of layers I–III (**d**) but no layer-specific change in the total number of NeuN-positive cells in the cortical layers (**e**). Pups with IVH had a reduction in synaptophysin-positive staining area as compared to controls (**f**) shown in a representative photomicrograph from the thalamus in (**g**) (scale bar = 25 μM). Data shown are median ± 95% CI. Evaluated with a two-way ANOVA and Sidak post hoc test. Control, *n* = 6, IVH, *n* = 6. *p < 0.05; **p < 0.01; ***p < 0.001.

In the neuron density assessment, IVH pups had a lower number of neurons overall in the five assessed regions (*p* = 0.008, two-way ANOVA) compared to controls (Fig. 4a, b). In the post-test, this was specifically apparent in the thalamus (*p* = 0.035) compared to controls. Focusing specifically on the cortex, guided by layer-specific staining of adjacent sections (Fig. 4c–e) we noted that there was no effect on the numbers of mature neurons each in layers I–III, IV, and V–VI (Fig. 4e). The total cortical depth in the IVH rabbits was marginally smaller but not significantly different from that in the controls (mean ± SEM) control, 1776.69 ± 245.39, *n* = 6 vs IVH, 1674 ± 188.29, *n* = 6). However, analysis of the width of each of the cortical layers, as a percentage of total cortical depth, revealed that there was an effect of IVH (*p* = 0.0289, two-way ANOVA, Fig. 4d). In the post-test, the depth of the upper cortical layers (I–III) was found to be significantly reduced in IVH brains compared to controls (*p* = 0.044; Fig. 4d). The IVH group animals also had a reduced density of presynaptic immunoreactivity (synaptophysin staining) across the six regions examined (*p* = 0.003, two-way ANOVA) compared to the controls (Fig. 4f, g).

### IVH altered the number, distribution and maturation of interneurons

**Fig. 5:**
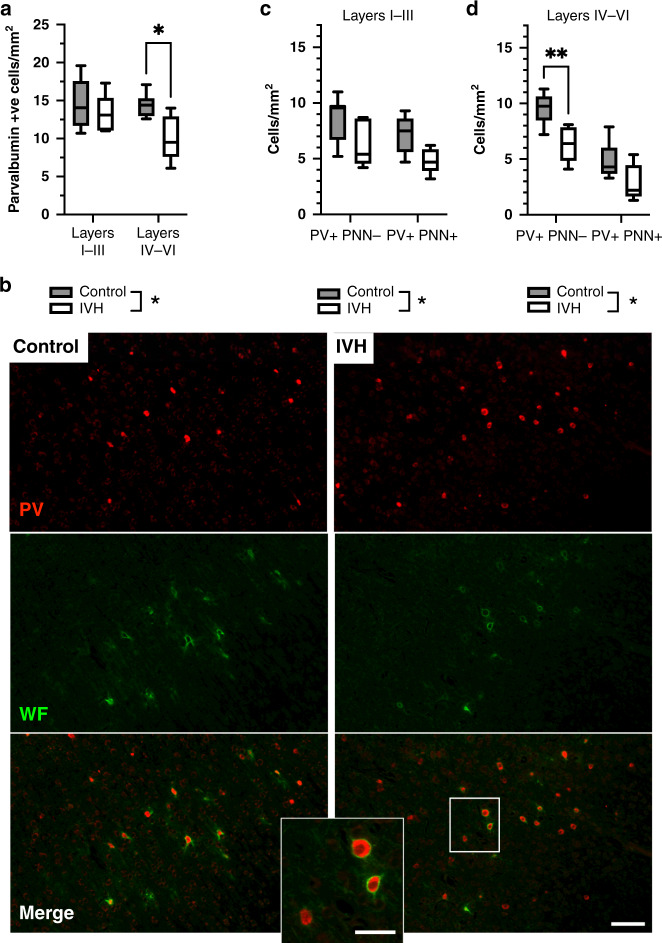
Interneuron development is altered in pups with IVH and PHVD. In pups with IVH vs controls, there was a reduction in the numbers of PV-positive interneurons in the lower cortical layers (IV–VI) (**a**) and there was a specific reduction in the PV-positive cells not co-positive for the perineuronal net (PNN) protein (**b**), There was no difference in PV-positive cells with or without PNN in the upper layers (layers I-III) (**c**), again in the lower cortical regions only as shown in **d**. Data shown are median + /− 95% CI. Control vs IVH evaluated with two-way ANOVA. Control, *n* = 6, IVH, *n* = 6. *p < 0.05. **p < 0.01. (Scale bar = low magnification 100 μm, high magnification inset = 50 μm).

Cortical layer markers (CTIP2/NECAB) were used to demarcate the upper (I–III) and lower (IV–VI) layers (as per Fig. 4c) and adjacent sections were stained for PV and WF for the PNN. The number of PV-positive interneurons in the cortical layers was significantly altered by IVH (*p* = 0.0244, two-way ANOVA), and the post hoc test highlighted a significant reduction in PV-positive cell number in layer IV–VI (*p* = 0.035, Fig. 5a). We also determined the total numbers of cells that were PV-PNN positive (PNN+) interneurons and PV-PNN negative (PNN−) interneurons (Fig. 5b–d). There was a significant effect of IVH on the number of PNN expressing PV interneurons in the upper cortical region (I–III, *p* = 0.0096, two-way ANOVA) but in the post hoc no specific location change (Fig. 5c). There was also a significant effect of IVH on the number of PNN expressing PV interneurons in the lower cortical regions (IV–VI; *p* = 0.0013, two-way ANOVA). The post hoc specifically localized this to a reduction in the numbers of PV + PNN− cells (*p* = 0.0073, Fig. 5d).

### IVH caused no change in GFAP but decreased the area coverage of IBA1

**Fig. 6:**
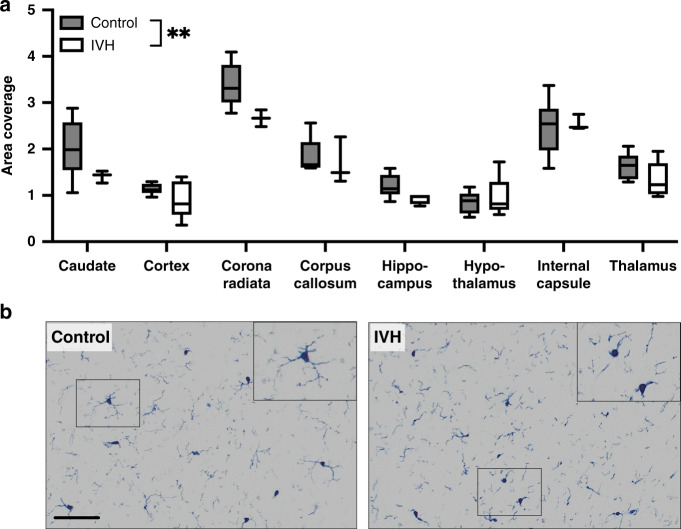
IBA1 area coverage was significantly decreased in pups with IVH. Pups with IVH had a reduction of IBA1 area coverage as compared to controls **a** as shown in representative photomicrographs of the cortical MBP staining in **b** (scale bar = 100 μM). Data shown are median ± 95% CI. Control, *n* = 6, IVH, *n* = 6. Evaluated with two-way ANOVA, **p < 0.01.

The number of GFAP-positive astrocytes did not significantly differ due to IVH, across the evaluated brain regions (nucleus caudate, thalamus, hypothalamus, hippocampus, or cortex) except the internal capsule showing the significant reduction of GFAP-positive cells in IVH pups compared to the controls (two-way ANOVA, data not shown). The IVH group animals had a reduced density of IBA1 immunoreactivity (microglia/macrophage marker) across the eight regions examined (*p* = 0.086, two-way ANOVA) compared to the controls (Fig. 6a, b).

## Discussion

This is the first small animal study on the long-term effects of IVH (out to PND 30) with subsequent PHVD development in a preterm animal model. IVH caused persistent changes in cortical structure, neuronal number, synapse density, and myelin. However, we could not detect any alterations in the neurobehavioral assessment. Most studies previously undertaken in models of preterm rabbit IVH have been focused on short-term outcomes, predominantly terminating at PND14.^20,21,27,35–48^

Survival in the preterm rabbit IVH model has previously not been described beyond PND14. However, in this study, most pup loss occurred during the first week of life, which agrees with previous findings.^20,27,38^ Compared to non-IVH preterm rabbit studies, we reported a lower overall survival within the first month of life (40% vs previous 56%.^32,49^) Also, we observed a reduced survival in female pups in the IVH group that has not been previously reported for preterm non-IVH pups.^31,49^ Survival based on sex has not been reported previously in short-term studies in the preterm IVH rabbit pup model. In human infants, it is male sex that is associated with higher mortality and major morbidity.^50^ Unfortunately, due to the high mortality in female pups, we could not undertake sex-specific analysis in this study, but we observed no difference in somatic growth indices. Importantly, using this data future studies will be powered to enable the capture of sex-related differences in outcome.

Our study is the first to apply rearing by a wet nurse in the preterm rabbit pup IVH model instead of gavage feeding. A wet nurse avoids the mechanical trauma caused by repeated insertion of the feeding tube, as well as stress and hypothermia and has previously been described in a preterm rabbit model of fetal growth restriction.^51^ The importance of breastfeeding in rabbit pups was emphasized in a recent study, where term pups reared by does display improved social interactions when compared to term-born gavage reared pups.^52^ Olfactory cues from lactating rabbit does are involved in breathing stimulation, distress response alleviation, attention, and directional response stimulation and may promote learning and social behavior.^53,54^

We undertook neurobehavioral testing in this study, because for children who develop PHVD, motor, co-ordination, and neurocognitive issues are frequent and do not decline with increasing age.^9–14^ Ours is the first study to evaluate neurobehavioral assessment at PND30. Previous work with this model at PND1–3 and PND14 demonstrated that pups with IVH have impaired neurobehaviour,^20,35,36,41,42,45–47^ including impaired motor functions and altered gait ability at P3^27^ and PND14.^20,35,36,41,42,45–47^ At this extended time point (P30), we were unable to detect any differences in motor, gait, coordination, or for global motor activity and exploration as an indicator of anxiety levels. We speculate that this absence of neurobehavioral deficit may stem from advanced recovery in the pup, which is also often observed after early-life injury in the rodent,^17^ combined with the additional positive impacts of the wet nurse on development, described above.^52–54^ That said, we performed the ORT, using a paradigm similar to that previously validated by us^30,31^ to induce learning in preterm rabbits raised by wet nurse (as for this study). Thus, the lack of clear effects on motor, coordination and neurocognitive functions may be related to a strong compensatory capacity of the animals with the later testing time point (compared to previous studies) and maturation per se. We also cannot exclude that with increasing age neurobehavioral injury may manifest and longer-term follow-up is also warranted. Altogether, further optimization of behavioral paradigms should be explored in rabbits to strengthen the utility of this model.

Oligodendrocyte maturational arrest and hypomyelination is a hallmark of brain injury associated with preterm birth in infants.^55,56^ We observed a global reduction in the area coverage for myelin caused by IVH, supporting several short-term studies showing disruption of oligodendrocyte maturation and the myelination process in the preterm rabbit IVH model.^20,35,36,41,45,46^ Myelination in term rabbit pups initiates at around PND4^20^ and reaches its peak at around PND18, with a delayed but more rapid increase in the myelination of fiber tracts in the internal capsule compared to the corpus callosum.^57^ Changes in myelination quantity and also structure is considered to be responsible for long-term neurological sequela in human infants^58^ and IHV led to altered myelin fiber orientation in an analysis relatively sensitive to subtle changes in MBP organization.^59^

Along with WM damage in children and adults who developed IVH, recent research has focused on gray matter (GM) damage. Alterations in thalamocortical networks and reduced GM volume are associated with adverse cognitive outcomes and behavioral disorders in IVH compared to controls.^60–62^ The results of our study showed a collection of subtle neuronal deficits, including that preterm rabbit pups with IVH had a global reduction in mature neuronal number, altered cortical layering, and an interneuronapathy. Recent data from Dohare et al. showed a significant reduction of SAT2B and CUX1 expressing neurons in the upper cortical layer (II–IV) in preterm IVH pups at PND14^47^ supporting our data of upper cortical layer thinning. However, with our count of total cell numbers we were unable to detect any differences in total neuronal number, despite the region being significantly thinner. Furthermore, preterm rabbits with IVH/PHVD had a reduced number of neurons in the nucleus caudate and thalamus, highlighting the involvement of GM in the pathogenesis of IVH/PHVD. Reduced neurogenesis in the thalamus and nucleus caudate has been reported in several preterm human post mortem studies,^63,64^ as well as in different animal models of neonatal brain injury.^65–67^

The effect of IVH on cortical PV interneurons in the preterm rabbit pup was assessed for the first time in our study. Cortical interneuron populations are vulnerable in preterm infants as they are still undergoing migration and maturation at the time of preterm birth.^68^ Specifically, IVH/PHVD group animals had a lower number of PV-positive PNN-negative interneurons in the deep layers. In agreement, studies evaluating injury related to preterm birth and inflammation^33^ and hypoxia–ischemia^69^ have reported fewer PNN-positive interneurons. The PNN is a key regulator of perisomatic input to PV interneurons and thus overall network activity, meaning that tight regulation of the PNN is needed for typical brain function.^70^ It might be that IVH/PHVD disrupts the maturation of PV-positive interneurons possibly due to the damage of the choroid plexus, where homeobox protein orthodenticle homeobox-2 (Otx2), involved in the expression of PNN, is released.

IVH also significantly reduced synaptophysin, a pre-synaptic terminal marker, supported by similar observations in a guinea pig model of intrauterine growth restriction (IUGR),^71^ a rat model of IUGR,^72^ and by observations of synaptic degradation in human cases of hydrocephalus.^73^ Very little is known about the pattern of synaptogenesis in the rabbit, except a single study of the retina indicating that synaptogenesis occurs from PND9, reaching a plateau at PND20.^74^ The decreased expression of synaptophysin may reflect disrupted synaptogenesis including altered processes of synaptic remodeling by microglia. Synaptic pruning is a key function of microglia during the later stages of brain development,^75,76^ such that depleting microglia can change the structural connectome of the developing mouse brain.^77^ We observed a reduction in the area coverage of IBA1, likely a loss of complexity due to ongoing inflammatory activation. We have made similar reports previously in a model of preterm brain injury in the mouse^78^ and these kinds of region-specific subtle changes has also been reported in a model of IUGR in the piglet.^79^ That there was a change in morphology was qualitatively supported when observing the stained sections, as the cell processes were less elaborate.

Our model of IVH in preterm rabbit pups carries a high translational value to the human situation, as it reflects a true preterm scenario, with the pups having underdeveloped lungs and gut, and being deprived of placentally derived trophic factors. This is reflected in the fact that preterm birth alone in this model induces brain injury reminiscent of that seen in preterm born infants.^26,49^

All pups in our study received glycerol and we did not include “sham” control animals to evaluate of the effects of glycerol. We used this experimental design because: (1) renal function tests to define the glycerol toxicity in this model found them to be within normal limits, except for a slight increase in blood urea nitrogen,^27^ (2) there is no evidence of inflammation, cell death, or neuronal degeneration in the forebrain of animals treated with glycerol without IVH vs the pups without glycerol treatment,^27^ and (3) glycerol is widely used clinically, both in adult and pediatric populations, to treat brain edema and increased intracranial pressure^80,81^ with no reported changes in brain levels glutamate, pyruvate, and lactate in people injected with glycerol.^81^ However, the use of glycerol injected controls ensure that any biochemical effects of this osmotic agent are controlled for in both groups.

Inherent high mortality in this model led to small sample sizes and reduced power for the detection of significant differences between groups and a sex-specific analysis. Although we wish to mention that the apparent increased vulnerability of females in this paradigm is in contrast to the typical “male disadvantage” reported for perinatal brain injuries.^82,83^ Ours is the very first data on sex-based survival reported in the glycerol-induced IVH model and many rabbit studies in other paradigms have not reported sex or there have been no differences in outcomes.^84–86^ However, one study of the impacts of preterm birth on rabbit brain delivery has reported that, compared with females, males have less damage in the hippocampus (assessed via fractional anisotropy) and greater numbers of neurons in the caudate nucleus (NeuN-positive cell number).^49^

There may also be a selection effect, introduced by the “healthiest” preterm pups surviving to the study endpoint while the pups with severe damage dying during the neonatal period masks possible differences potentially present in the original sample size. This is an unavoidable phenomenon, also occurring in long-term follow-up studies of infants where early mortality is greatest for infants with the most severe IVH.

This study introduces important information for the field. For the first time, we report the long-term effects of IVH leading to PHVD in a preterm animal model that include alterations to myelination, including organizational changes, decreased mature neuron and presynaptic terminal densities, altered cortical organization, and altered PV-positive interneurons number and maturation. The lack of corresponding behavioral deficits may reflect the need for improved behavioral testing paradigms in this translationally valuable IVH model. These fundamental findings are important for furthering our understanding of mechanisms leading to WM and GM damage ultimately causing neurodevelopmental impairment in infants.

## Supplementary information

Supplementary Material

## Data Availability

The datasets generated during and/or analyzed during the current study are available from the corresponding author on reasonable request.
