# Supplementary material for: Severe intraventricular hemorrhage causes long-lasting structural damage in a preterm rabbit pup model
Source: Pediatr Res. 2022 May 3;92(2):403–14. doi: 10.1038/s41390-022-02075-y (PMC9522590; doi:10.1038/s41390-022-02075-y)
Supplement: Supplementary file 1 — Supplementary Material [file 41390_2022_2075_MOESM1_ESM.pdf]

Supplementary methods

Animals

The study was approved by the Swedish Animal Ethics Committee in Lund (dnr. M 2-16). We used the preterm rabbit pup model of glycerol-induced IVH as previously described [1,2]. Seventy-seven preterm rabbit pups of both sexes from 12 different litters were included in the study. A half-breed between the New Zealand White and Lop was used (Christer Månsson, Löberöd, Sweden). The rabbit does were sedated with intravenous propofol (5 mg/kg, Primen Pharmaceuticals Oy, Helsinki, Finland) and pups were delivered via cesarean section at post-conceptional day 29 (term = 32 days). The pups were dried and rubbed to stimulate breathing, marked for identification with a subcutaneous microchip (SweVet Piab Microchip, 1.4 × 8 mm, Sjöbo, Sweden), weighed, handfed 100 ml/kg of bovine colostrum (Whole Colostrum, ColoDan, Denmark) using a 3.5 Fr feeding tube (Vygon, Ecouven, France) and placed in a closed infant incubator set to a temperature of 32°C. At 3 h of age, all pups received intraperitoneal (i.p.) injection of 50% (*v/v*) sterile glycerol (6.5 g/kg; Teknova, Hollister, CA, USA) to induce IVH. Thereafter, pups were randomly allocated (https://www.random.org/) to a wet-nurse doe for the remainder of the experiment. The wet-nurse rabbit had given birth vaginally to pups at term within 24 hours of cross-fostering and all but two of her term offspring were replaced with preterm pups. Pups were weighed daily for 7 days, thereafter at postnatal day (PND) 11, and after that every 6 days. Bi-parietal measurement was performed weekly. The study endpoint was PND 33, which corresponds roughly to brain development of a year-old toddler [3].

Sex determination

Fresh skin tissue was collected at harvesting for DNA isolation using DNeasy Blood & Tissue Kit (Qiagen, Hilden Germany). Amplification of SRY fragment was employed in 30 cycles as follows: initial denaturation step 95 °C 3 min followed by 95 °C 30 sec, 57 °C 30 sec, 72 °C 1 min with last step 72 °C 10 min. Left primer: TGCAATACAGGAGGAACACG, right primer: AGCAAACTGTCGCTCTTCTG. The PCR product, 299 bp, were analyzed by electrophoresis in 1.7% agarose gel with SYBR safe DNA gel stain (Invitogen) and visualized by Image Lab software (BioRad).

Neurobehavioral examination

Muscle tone examination was done by active flexion and extension of forelegs and hind legs (score 0 to 3). The righting reflex was measured by the capacity of and time taken for preterm rabbits to turn to a supine position when placed prone. Gait and coordination were examined by testing the preterm pups' ability to hold their position when placed on a 60% incline slope. The test was conducted on a white rectangular plastic surface (70x60 cm) placed at 60° inclination. The pup was placed at the highest end of the surface and the latency to slide down the slope was measured. The gait was assessed by a scoring system from 0 (no locomotion) to 5 (normal walking).

The open field (OF) test and the object recognition task (ORT) were conducted in a square arena (140 cm × 140 cm) enclosed by 40-cm high plastic walls with a standardized light intensity across the arena, details below. The arena was cleaned with a 10% ethanol solution after each session to eliminate any olfactory cues. The ORT was undertaken at distinct intervals. Tests were

executed between 8 a.m. and 3 p.m. on different 4 consecutive days in the following sequence: OF; ORT habituation; ORT 30 min inter-trial interval; ORT 240 min (outlined below).

Open field test

The rabbits were taken from their cage one by one cloaked in a clean cloth and introduced in the top right corner of the arena. The arena was divided into three sub- zones: the center, the outer margin adjacent to the wall - “peripheral zone”, and the area in between - the “intermediate zone.” Latency before the rabbit left the entry point and time passed in the respective area during the 5 minutes were measured.

Object recognition task

Two objects were placed within the arena, 60 cm apart and 40 cm from the walls of the arena. The weight of the selected objects did not allow the rabbits to move or to jump and sit on the objects. Rabbits were taken from their cage cloaked in a clean cloth and transferred into a smaller cage for 5 min (L x W x H = 42cm x 19cm x 27cm) after which time each rabbit entered into the arena in one of the corners. The ORT included a familiarization phase (T1; 5 min) and a trial phase (T2; 5 min), divided by an inter-test interval of 5-, 30- and 240-min on testing days 2, 3, and 4, respectively. The first two sessions were counted as familiarization/training. In the familiarization phase (T1; 5 min) the rabbit could freely explore 2 identical items. Then the animal was taken out from the arena and replaced in its cage for either 5, 30, or 240 min. Prior to the test phase (T2; 5min), one of the items was substituted with a clean identical object, while a novel, distinct object, replaced the second item. The position of the novel object (right or left) was randomized. Each item was cleaned with 10% ethanol prior to and following each session. Exploration of the objects throughout T1 and T2 was specified as sniffing or touching while

pointing towards either item within a distance of <2 cm. Exploration indices "E1" and "E2" were calculated as the cumulative time to explore the objects during T1 and T2, respectively. For T2 only, "D1" reflected the difference in time exploring the old and novel object whereas "D2" (discrimination index) was calculated as (D1/E2). Rabbits were excluded from the analysis in case of absent exploration of any object during T1 or T2. Animal position was automatically registered by a PC connected to a video camera, installed 250 cm over the field. Data was analyzed with the Video Tracking Software (SMART, Panlab SL, Barcelona, Spain). The authors were blinded to animal group assignment throughout testing, data gathering, and analysis.

Tissue collection

Following neurobehavioral assessment, rabbits were sedated with intramuscular ketamine (35 mg/kg, Intervet International B.V, Boxmeer, The Netherlands) and xylazine (6 mg/kg, VM Pharma AB, Sweden). Pups were then transcardially perfused with phosphate buffer saline (PBS, pH 7.4, containing 0.01% of heparin), followed by perfusion with freshly prepared 4% paraformaldehyde (PFA, VWR Chemicals, Leuven, Belgium, buffered with PBS, pH 7.4). The brains were post-fixed by immersion in 4% PFA for a total of 24h. Brains were dehydrated in a graded ethanol serial (70–99.99%) and Xylene (100%) and embedded in paraffin blocks. Coronal sections (5 μm) were cut on a rotating microtome (Microm HM 360, Microm International GmbH, Walldorf, Germany), and sections were collected on microscope slides (SuperFrost Plus, Thermo Scientific/Gerhard Menzel B.V. & Co., Braunschweig, Germany).

Immunohistochemistry

Sections were de-paraffinized and rehydrated and heat induced epitope retrieval (citrate buffer, pH 6 + 0.04% Tween 20, at 95°C for 20 minutes) undertaken. Sections were incubated with hydrogen peroxidase (0.3 %, in 0.1M PBS) for 10 min and then in 0.1M PBS containing 0.05% Triton X-100 (TX) and 1% bovine serum albumin (PBS-BSA-TX), for 30 min at room temperature. Primary antibodies listed in Table 1 were diluted in PBS-BSA-TX and applied for 16 h, at 4 °C in a humidified chamber: As antibody specificity controls, in adjacent sections the primary antibody incubation was excluded. Sections were rinsed in PBS containing TX (0.05%, PBS- TX) and incubated with horseradish peroxidase (HRP) conjugated secondary antibodies, see table X, for 30 min at room temperature in a humidified chamber. Sections were then rinsed in PBS-TX and incubated in a 0.1M PBS solution containing diaminobenzidine (DAB, 0.5 mg/ml) and hydrogen peroxidase (0.1%), for 10 min at room temperature. Following rinses in 0.1M PBS sections were counterstained with hematoxylin and dehydrated in a grade graded ethanol, then xylene before mounting and coverslipping in Pertex, (Histolab, Gothenburg, Sweden).

Immunofluorescence

A standardized IF protocol was conducted as previously described [4] including no primary antibody controls. In brief, slides were cleared with histolene and rehydrated incubated in 1xPBS (3x5 min/wash), and antigens retrieved with citrate buffer (10mM, pH 6.0) at 95oC for 20 minutes. After washing (PBS) sections were then exposed for 20 min to a 0.1% Sudan Black block (Sigma Aldrich, 199664) diluted in 70% EtOH to reduce autofluorescence, as previously [5]

and then blocked further in 5% normal goat serum (NGS; Thermoline Scientific, 10000C) 0.2% Triton X-100 (Sigma Aldrich, 10789704001) solution diluted in 1xPBS for 1 h at room temperature. Slides were incubated in humidified chambers overnight at 4℃ with the antibodies listed in Table 1. Slides were washed (PBS), and for staining perineuronal nets (PNN) Wisteria Floribunda lectin was added for 1 h at room temperature. The diluted fluorescent secondary antibodies (Invitrogen) were added to sections for 1 h at room temperature, Table 1. After washing, slides were incubated in 4,6-diamidino-2-phenylindole (DAPI; 1:1000; Invitrogen D21490) diluted in 1xPBS and 0.1% Triton X-100 for 15 min, washed (3x5 min PBS) and coverslipped (Sigma Aldrich, F4680). Antibodies and lectins were diluted in 1xPBS, 2.5% NGS, and 0.1% Triton X-100. PV and WFL staining were conducted in sections immediately adjacent to CTIP2 and NECAB1.

Analysis of immunohistology

Slides were scanned to obtain digital images (Hamamatsu, NanozoomerS60, Hamamatsu, Japan) under fixed lighting conditions and regions of interest (ROI) were manually annotated using Hamamatsu NDP.view 2 software and extracted as Jpg images and converted to 8 bit in Fiji/ImageJ (http://fiji.sc). NeuN positive cells were calculated with Fiji by the default auto-threshold segmentation method and watershed separation following primary smoothing or were manually counted if the separation of cells was not achieved. Small objects, not of neuronal cell body size, were removed. The number of identified cells was divided by the whole area for neuron density assessment. ROI´s with GFAP, synaptophysin, and MBP labeling were calculated using a fixed threshold (relative to background) and the relative area (positive area/overall area) was calculated. The mean of the left and right regions was used for statistical analysis. We also

analysed directionality and organization of the MBP staining using Fiji (https://doi.org/10.1088/1758-5090/aa6204). MBP was assessed in the middle third of a fixed-width image of the cortical plate to focus on the region with the greatest number of ascending fibers. Regions of interest were extracted using the NDP.view 2 viewer and Photoshop with scale and resolution fixed at all points across the analysis.

Analysis of immunofluorescence

Within 14 days of IF staining, digital images were attained at x20 magnification using the VS120 Olympus Virtual Slide Microscope (Olympus Life Science, VIC, Australia). Image analysis was performed using CellSens Dimension software (Olympus). For both stains, regions of interest (ROI's) were extracted from the medial and lateral sides of the parietal cortex from both the left and right hemispheres. To standardize ROI extractions, the medial parietal cortex was delineated by the terminal region of the cingulate cortex, and the lateral parietal cortex demarcated by a marking a line passing vertically through the cortex meeting the most lateral point of the hippocampal gyrus.

For cortical layering analysis, layer measurement was standardized by drawing a polyline (segmented at one point in the approximate center) at the start of layer I that spanned 2mm of the cortex. The line was duplicated and lowered until reaching 3 intense NECAB1+ cells that represented the start of layer IV and this distance represented the length of layers I-III. Again, the segmented line was duplicated and lowered until it reached cortex absent in NECAB1+ cells representing the end of layer IV. This was repeated for measuring layers V-VI, where the end of layer VI was defined by the absence of CTIP2+ cells. Total cortical depth (layers I-VI) was also

measured and all measurements were conducted twice on opposing sides of the segmented 2mm line.

For interneuron analysis, upper and lower cortical regions were defined as layers I- III and IV-VI, respectively, as described previously [4]. ROIs were extracted as described for cortical layering analysis, and layers were segregated according to the subject-specific layer measurements. Immunoreactive cells were counted manually by a blinded observer using the CellSens Dimension counter tool. PV+ cells were identified as having a defined cell body within the section, and WFL+ PNN+ were defined as an entire halo encompassing a PV+ interneuron. All PV+ interneurons were counted in upper and lower layers then an alternate-colored marker was used to count PV+ PNN+ cells. Tissue was excluded if it was damaged or had an excessive background or non-specific staining.

References

1. Chua, C. O. *et al.* Consequences of intraventricular hemorrhage in a rabbit pup model. *Stroke* **40**, 3369–3377 (2009).
2. Sveinsdottir, S., Cinthio, M. & Ley, D. High-frequency ultrasound in the evaluation of cerebral intraventricular haemorrhage in preterm rabbit pups. *Ultrasound Med. Biol.* **38**, 423–431 (2012).
3. Dutta, S. & Sengupta, P. Rabbits and men: relating their ages. *J. Basic Clin. Physiol. Pharmacol.* **29**, 427–435 (2018).
4. Stolp, H. B. *et al.* Interneuron development is disrupted in preterm brains with diffuse white matter injury: Observations in mouse and human. *Front. Physiol.* **10**, 955 (2019).
5. Sun, Y. *et al.* Sudan Black B Reduces Autofluorescence in Murine Renal Tissue. *Arch. Pathol. Lab. Med.* **135**, 1335–1342 (2011).
